# Post-exercise Recovery: Cooling and Heating, a Periodized Approach

**DOI:** 10.3389/fspor.2021.707503

**Published:** 2021-09-01

**Authors:** Robin T. Thorpe

**Affiliations:** ^1^Football Exchange, Research Institute for Sport and Exercise Sciences, Liverpool John Moores University, Liverpool, United Kingdom; ^2^College of Health Solutions, Arizona State University, Phoenix, AZ, United States

**Keywords:** recovery, fatigue, exercise, injury, cooling, heating, muscle damage (DOMS), periodization

## Rising Importance of Recovery

Recovery is regarded as a multifaceted (e.g., physiological, psychological) restorative process relative to time and modulated by external load, individual response to stress, and often dictated by external athletic competition and demand (Kellmann et al., 2018). The increasing physical demands of athletic competition, particularly, team sports (Barnes et al., 2014), involving high fixture frequency, has further exacerbated the physical and mental load placed on athletes (Ekstrand et al., 2018). Athletes are now routinely exposed to longitudinal demands with, in some cases, only 48 h of recovery time between competitions. Fatigue may be defined as “an inability to complete a task that was once achievable within a recent time frame” (Pyne and Martin, 2011; Halson, 2014) and derived from central and/or peripheral origins. Recovery time between successive competitions may be insufficient to allow athletes to fully regenerate leading to fatigue, which may increase the risk of under-performance, non-functional overreaching, injury, and illness (Dupont et al., 2010; Bengtsson et al., 2013). Demands are further increased in athletes competing in continental leagues, play-off phases, international tournaments, and are further aggravated in circumstances such as the English Premier League that does not include a winter break (Ekstrand et al., 2018) or in recent times the effect of the COVID-19 pandemic (Seshadri et al., 2021). Increased athlete training and competition availability as a result of a reduction in injuries, substantially improves the likelihood of success of an individual or team (Hägglund et al., 2013). Changes in injury occurrence also have a significant impact, particularly, financial implications (team underachievement and player salaries) of sporting organizations due to injury-related decrements in performance (Eliakim et al., 2020). Growing demands and the rising importance of improving recovery have also prompted athletes to inclusively invest in further bespoke personal support in an attempt to accelerate recovery.

## Evidence and Practice: a Confusing Landscape

A certain degree of fatigue, resulting in functional overreaching, is required to mediate adaptations to training, which drive performance enhancement (Noakes, 2000). However, excessive fatigue through insufficient recovery may increase susceptibility to non-functional over-reaching, injury, and illness of the players (Nimmo and Ekblom, 2007). Fatigue can be compensated with recovery strategies which serve to restore homeostasis on a physiological and psychological level (Kellmann, 2002). Researchers and practitioners alike have investigated the efficacy of commonly utilized interventions to combat the deleterious effects of athletic training and competition (Barnett, 2006; Howatson and van Someren, 2008; Nédélec et al., 2012; Dupuy et al., 2018). A recent investigation (Altarriba-Bartes et al., 2020) reviewing commonly used recovery strategies in professional soccer found that all teams were utilizing at least one recovery strategy following games; however, the range of interventions used was substantially different between teams with water immersion (cold and hot), massage, and foam rolling representing 74, 70, and 57% respectively (Altarriba-Bartes et al., 2020).

It is imperative that the origin of fatigue is understood in order to most effectively return the human body to homeostasis following exercise. Furthermore, an understanding of the origin of fatigue may help with tailoring an appropriate recovery strategy to enhance the accelerated return to homeostasis. Recovery time from training-induced stress may differ within and between the different organismic systems of the human body (Kellmann et al., 2018). The increased focus on athlete recovery within professional sport has naturally been followed by many scientific investigations attempting to understand the efficacy of a range of commonly performed strategies (Howatson and van Someren, 2008; Leeder et al., 2012). However, few studies have been able to demonstrate the efficacy of strategies improving recovery in athletes following training or competition (Bieuzen et al., 2013; Hill et al., 2014; Dupuy et al., 2018; Davis et al., 2020).

Much of the positive evidence for recovery strategies lies with an enhanced perceptual outcome of recovery, often attributed to an athlete's belief in the modality or the placebo effect (Broatch et al., 2014; Wilson et al., 2018). Indeed, evidence exists whereby recovery strategies have not improved fatigue levels further than that of the placebo effect (Cook and Beaven, 2013; Broatch et al., 2014; Malta et al., 2019). Research has traditionally focused on administering one recovery intervention at a time, whereas in the applied setting athletes are more likely to administer multiple interventions in varying sequences due to the many strategies available, of which many lack efficacy (Costello et al., 2016; Davis et al., 2020; Skorski et al., 2020). Although extensive, the existing literature base investigating recovery strategy efficacy still lacks clarity and directional influence for practitioners and athletes alike. Much of the data involves study designs investigating changes in physical performance and perceptual or muscle damage markers following an exhaustive protocol or athletic competition (Leeder et al., 2012; Davis et al., 2020). These methodological variances alongside less realistic laboratory protocols detached from contextual performance and investigation of only the acute recovery response (0–72 h), including sub-elite subject cohorts may be some of the reasons why inconclusive data exists (i.e., sole strategies performed across entire recovery continuum), indirectly, creating confusion for practical application. Movement toward a more periodized research design approach has occurred where multiple strategies have been assessed in an attempt to improve recovery (Martínez-Guardado et al., 2020; Pooley et al., 2020). Reasons for applying multiple modalities may arise from the fact that athletes are now exposed to a variety of strategies and professional philosophy, proposed to enhance recovery, rather than a physiology-based rationale. Athletes performing multiple strategies rather than a singular modality may be a step forward; however, a more critical, evidence-based reasoning for the application of periodizing varying strategies is required. A better understanding of the exact physiological systems and mechanisms of fatigue may provide a clearer landscape into unraveling recovery from exercise, performance, and injury.

## Matching the Stress and Intervention: Monitoring-Based Practice

Physical demands of both individual and team sports involve varying contributions of metabolic and mechanical stress to tissue. Mechanical stress deriving primarily from eccentric contractions results in a temporary reduction in muscle function, an increase in intracellular proteins in the blood, an increase in perceptual muscle soreness, and evidence of swelling (Howatson and van Someren, 2008). Thereafter, secondary damage is linked to the subsequent inflammatory response and macrophage and neutrophil infiltration which, further, in isolation compromise the mechanically stressed area (Merrick, 2002). Metabolic factors such as reductions in adenosine triphosphate (ATP), creatine phosphate, glycogen (Krustrup et al., 2006), and pH (Brophy et al., 2009) may also induce fatigue following exercise. Biochemical changes in electrolytes and calcium may also have negative effects alongside hypoxia at the muscle cell level contributing to metabolic fatigue. Mechanical stress and/or metabolic fatigue may also contribute to neuromuscular cost *via* altered muscle potassium and pH levels (Tee et al., 2007) and excitation contraction coupling, respectively (Jones, 1996). Environmental factors and exercise-induced heat generation (Arbogast and Reid, 2004), which increases the concentration of nicotinamide adenine dinucleotide phosphate oxidase within the muscle fiber resulting in an increase in the production of reactive oxygen species from the mitochondria and from the infiltrating inflammatory cells (Powers and Jackson, 2008) further exacerbating potential mechanical damage. The variance in physiological origin associated with exercise and competition infers that it is illogical that a single recovery strategy and/or a generic “one size fits all” approach would accelerate each of the systems discussed (Minett and Costello, 2015). Evidence exists where a singular temperature-based strategy applied locally to the quadriceps over the entire recovery continuum failed to further accelerate recovery beyond the acute period (0-72 h), *albeit*, following severe marathon running–derived mechanical and metabolic stress (Kwiecien et al., 2020a,b). Moreover, Petersen and Fyfe (2021) suggested, from a chronic perspective, long-term application of a singular intervention may have disadvantages relating to adaptation (Petersen and Fyfe, 2021). It appears that a binary perspective to recovery has arisen within the literature, which in turn may have influenced the applied setting. Alternatively, a framework where strategies are periodized to match the individual symptoms, organismic fatigued system, external load or the response to stress may be a more preferred approach (Thorpe et al., 2017; Kellmann et al., 2018). Indeed, monitoring of recovery or the response to load may provide insights into the exact physiological stress an athlete is currently experiencing. A recent review stated that the quantification of physiological stress *via* athlete response outcome measures, athlete self-report, heart rate-derived autonomic nervous system, neuromuscular functional jump/eccentric/concentric/isometric protocols, biochemical/immunological/endocrine, and joint range of motion could improve practical prescription of modalities in enhancing recovery (Thorpe et al., 2017). For example, assessing changes in perceived muscle soreness or the autonomic nervous system *via* heart rate-derived metrics (heart rate variability and/or heart rate recovery) may establish whether or not an athlete is experiencing symptoms associated with mechanical damage (Dupuy et al., 2018), thus a gateway to understanding and quantifying which strategies may be most appropriate for improving this fatigued system. Attention ought to be prioritized to framework strategies that match the associated physiological stress along the recovery continuum in a systematic manner.

## Temperature-Derived Approach: Periodizing Cooling and Heating

Beyond sleep, nutrition, and hydration, recent work has focused on the application of various temperature-based modalities in an attempt to accelerate recovery (McGorm et al., 2018; Kwiecien and McHugh, 2021). Indeed, among the vast array of recovery strategies commonly used by athletes, temperature-based modalities have shown the most promise, although, still the data are inconclusive (Jakeman et al., 2009; Stanley et al., 2012; Broatch et al., 2014). One of the most common recovery strategies used is cryotherapy, or the application of cooling (Altarriba-Bartes et al., 2020). Cooling has been performed for decades in relation to injury, and intuitively, transferred to recovery from exercise in more recent times. Topical cooling, cold water immersion, whole body cryotherapy, and more recently phase change material are most commonly used in both the clinical and professional sports settings (Kwiecien and McHugh, 2021). The mechanistic response between these modalities has been shown to differ and in some circumstances provides a completely different physiological effect (Mawhinney et al., 2017; Kwiecien and McHugh, 2021). The ultimate objective for cooling is to reduce deep muscle temperature, in an attempt to favorably reduce blood flow and metabolism at the affected muscle site, in an effort to diminish the secondary damage phase (Merrick, 2002). A recent review suggested that repeat application or elongating cooling time would lead to the most advantageous results in reducing deep muscle temperature, in turn, the proliferation of the secondary damage phase (Kwiecien and McHugh, 2021). Importantly, local changes in muscle temperature (cooling or heating) may influence enzymatic activity and effect rates of intramuscular glycogen resynthesis (Cheng et al., 2017). Indeed, research has also investigated the effects of heating regarding performance, adaptation, and to a lesser extent recovery (McGorm et al., 2018). Heat therapy including hot water immersion has not been widely investigated in terms of athletic recovery, although, anecdotally performed frequently in athletes across many sports (Altarriba-Bartes et al., 2020). Data exists supporting heating for stimulating local blood supply and metabolism in tissues, and emerging evidence indicate that heat activates more specific molecular events, including changes in gene expression, anti-inflammatory and antioxidant effects, glycogen resynthesis, mitochondrial biogenesis, heat shock protein expression, and cellular healing (Hoekstra et al., 2008; McGorm et al., 2018; Nadarajah et al., 2018). Data from animal and human studies have shown metabolic-based recovery to be accelerated following heat application which in turn modified the release of tetanic [Ca^2+^] and glycogen resynthesis rates compared to cooling (Cheng et al., 2017). Considering the existing evidence of the possible recovery kinetics to both cooling and heating, it appears that increasing or decreasing tissue temperature may provide advantageous responses at varying points on the recovery continuum, which are associated to mechanical damage and metabolic fatigue.

## A Practical Guide

**Figure 1:**
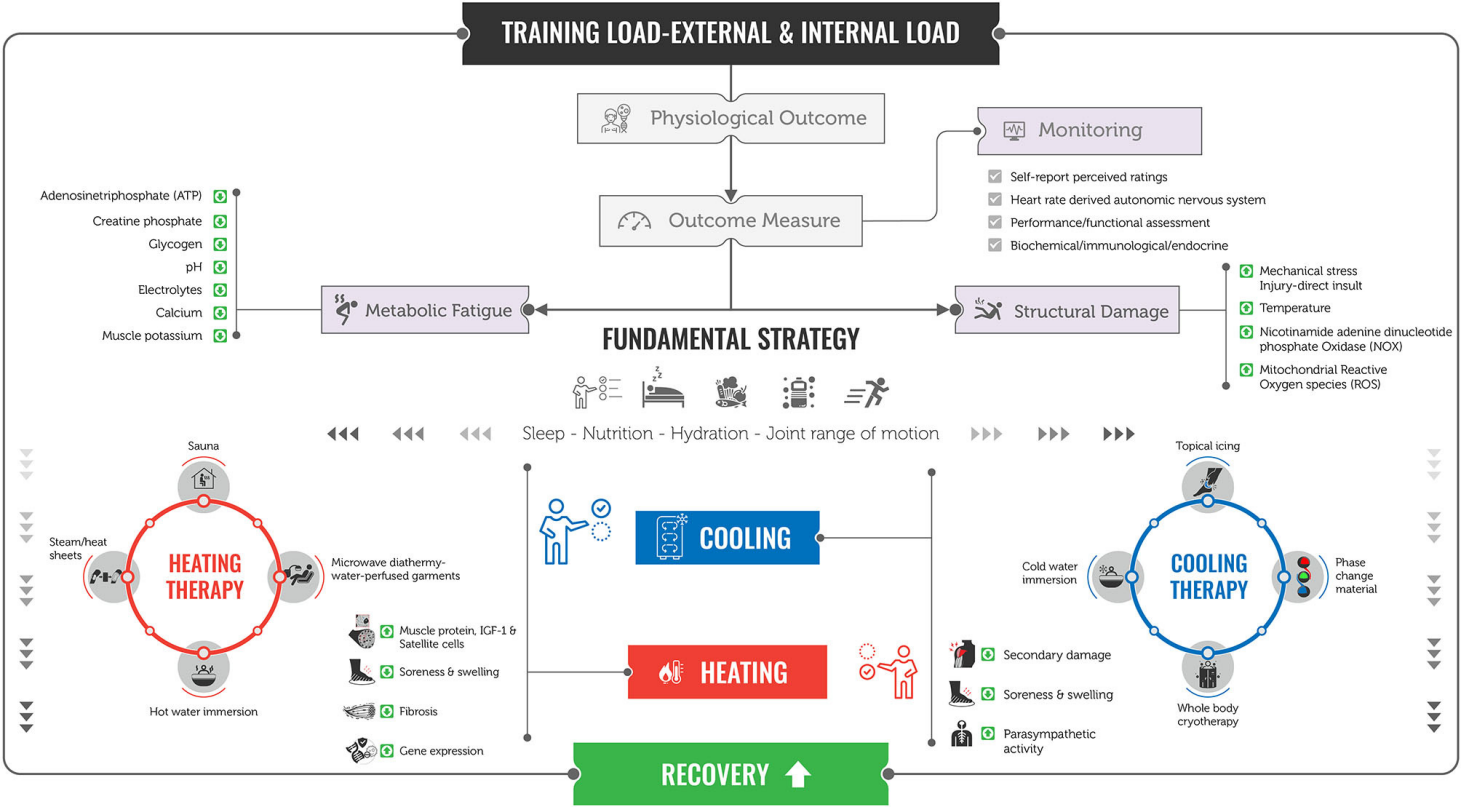
Practical framework to enhance recovery in athletes. Following external and internal load–stress response outcome measures, including, self-report [soreness (DOMS), perceived fatigue, sleep quality]; heart rate-derived autonomic nervous system [heart rate variability (HRV), heart rate recovery (HRR), submaximal heart rate (HRex)]; performance/neuromuscular functional assessment (force–time jump, eccentric, concentric, isometric protocols); and biochemical/immunological/endocrine (creatine kinase, IL-6, C-reactive protein) may be used to distinguish limiting components of physiological fatigue systems {structural damage [mechanical stress, direct insult, *increases in* temperature and nicotinamide adenine dinucleotide phosphate oxidase (NOX), and mitochondrial reactive oxygen species (ROS)] and metabolic fatigue [*fluctuations in* adenosine triphosphate (ATP), creatine phosphate, glycogen, pH, electrolytes, calcium, and muscle potassium]} following exercise and injury. Following an initial fundamental strategy (*optimal* sleep, nutrition, hydration, and joint range of movement) the use of cooling (topical icing/phase change material, cold water immersion, whole body cryotherapy) and heating (sauna, microwave diathermy water-perfused garments, hot water immersion or steam/heat sheets) strategies may be used systematically and independently to match the stress to assist in alleviating structural damage (secondary damage, soreness, swelling, and parasympathetic reactivation) and/or metabolic fatigue (muscle protein, IGF-1, satellite cells, soreness, swelling, fibrosis, and gene expression), respectively.

An array of different strategies are used by athletes in an attempt to alleviate the deleterious symptoms associated with exercise and competition (Nédélec et al., 2013; Altarriba-Bartes et al., 2020). However, there is a lack of consensus in how to design and prescribe strategies in improving the multifactorial systems of recovery. It appears that the first and most critical physiological event to attempt to mediate is the secondary damage phase shortly following mechanical damage. The latest evidence suggests that prolonged cooling is the most suitable intervention (Kwiecien and McHugh, 2021). Cooling *via* water immersion (in some cases multiple exposures) or local phase change material has been shown to have the most effective results in reducing tissue temperature (Mawhinney et al., 2017; Kwiecien et al., 2020b). Hereafter, and to promote removal and enhanced transportation of metabolic byproducts, and possible modulation of cellular healing, hemodynamics and substrate resynthesis (McGorm et al., 2018) heating is preferred *via* sauna microwave diathermy, water-perfused garments, hot water immersion, or steam/heat sheets (Hyldahl and Peake, 2020). This proposed framework (Figure 1) may be individualized based on the proportionate expense of mechanical and metabolic fatigue and whereby increasing or decreasing tissue temperature beyond purely an individualized approach, and when response to load/fatigue monitoring is limited (Thorpe et al., 2017), periodizing strategies to not only consolidate recovery across a training period but also in an attempt to enhance adaptation is proposed. Indeed, sequencing cooling strategies following endurance dominant stress or heating strategies following strength-derived stress may induce advantageous gene expression-related adaptations (Allan et al., 2017; Cheng et al., 2017; Hyldahl and Peake, 2020). The role of cooling and heating modalities should be chosen in reflection of external physical demand and matched accordingly to negate any contraindicative effect to adaptation interactions (Peake et al., 2020).

There is a clear physical and mental stress induced by exercise, competition, and acute injury. A unique physiological and immunological cascade then ensues. Identifying the different and proportionate mechanistic alterations is paramount in order to mitigate against further reduced performance, injury, and illness risk. Prioritizing sleep, rest, nutrition, hydration, and joint range of motion during this phase is fundamental, thereafter, recovery interventions should be considered that alleviate the particular physiological stress incurred at any given time point on the recovery continuum (Kellmann et al., 2018). Reducing tissue temperature *via* cooling has shown to mediate secondary damage derived from mechanical stress (Merrick, 2002), whereas heating has been shown to enhance tissue temperature, blood flow, and metabolism alleviating metabolic-associated fatigue (McGorm et al., 2018). Identifying origins of fatigue *via* the use of practical monitoring processes is recommended for individualization of recovery strategy prescription (Thorpe et al., 2017). In the absence of fatigue monitoring, a generic approach in which reducing secondary damage *via* cooling as the initial strategy followed by heating once the inflammatory cascade diminishes is recommended because of the timeline and functional detrimental properties of this process. The utilization of cooling and heating to navigate and facilitate the associated perturbations may be considered appropriate to accelerate recovery *via* the different physiological demands in athletes. A periodized, systematic recovery process matching appropriate thermoregulatory strategies to associated physiological systems should be considered as a framework to enhance recovery in athletes.

## Author Contributions

The author confirms being the sole contributor of this work and has approved it for publication.

## Conflict of Interest

The author declares that the research was conducted in the absence of any commercial or financial relationships that could be construed as a potential conflict of interest.

## Publisher's Note

All claims expressed in this article are solely those of the authors and do not necessarily represent those of their affiliated organizations, or those of the publisher, the editors and the reviewers. Any product that may be evaluated in this article, or claim that may be made by its manufacturer, is not guaranteed or endorsed by the publisher.
